# Early Evolution of Conserved Regulatory Sequences Associated with Development in Vertebrates

**DOI:** 10.1371/journal.pgen.1000762

**Published:** 2009-12-11

**Authors:** Gayle K. McEwen, Debbie K. Goode, Hugo J. Parker, Adam Woolfe, Heather Callaway, Greg Elgar

**Affiliations:** School of Biological and Chemical Sciences, Queen Mary University of London, London, United Kingdom; Tokyo Institute of Technology, Japan

## Abstract

Comparisons between diverse vertebrate genomes have uncovered thousands of highly conserved non-coding sequences, an increasing number of which have been shown to function as enhancers during early development. Despite their extreme conservation over 500 million years from humans to cartilaginous fish, these elements appear to be largely absent in invertebrates, and, to date, there has been little understanding of their mode of action or the evolutionary processes that have modelled them. We have now exploited emerging genomic sequence data for the sea lamprey, *Petromyzon marinus*, to explore the depth of conservation of this type of element in the earliest diverging extant vertebrate lineage, the jawless fish (agnathans). We searched for conserved non-coding elements (CNEs) at 13 human gene loci and identified lamprey elements associated with all but two of these gene regions. Although markedly shorter and less well conserved than within jawed vertebrates, identified lamprey CNEs are able to drive specific patterns of expression in zebrafish embryos, which are almost identical to those driven by the equivalent human elements. These CNEs are therefore a unique and defining characteristic of all vertebrates. Furthermore, alignment of lamprey and other vertebrate CNEs should permit the identification of persistent sequence signatures that are responsible for common patterns of expression and contribute to the elucidation of the regulatory language in CNEs. Identifying the core regulatory code for development, common to all vertebrates, provides a foundation upon which regulatory networks can be constructed and might also illuminate how large conserved regulatory sequence blocks evolve and become fixed in genomic DNA.

## Author Summary

Recent comparative analyses of vertebrate genomes has resulted in the identification of highly conserved non-coding sequences near genes that coordinate early development. Many of these sequences can activate gene expression and are thought to be important regulatory elements. Surprisingly, a large set of these long, near-identical sequences is found in every jawed vertebrate, including sharks, yet almost completely absent in non-vertebrates. This study looks for this set of sequences in the lamprey, a representative of our most distant vertebrate relatives, in order to determine when and how such a large set of important non-coding regulatory sequences became established in the genome. Although the lamprey divergence is only a little older than the divergence of cartilaginous fish (including sharks), relatively few, and considerably shorter, conserved non-coding sequences are identifiable. Nevertheless, these shorter lamprey sequences are capable of driving gene expression in a precise spatial pattern in zebrafish embryos in the same way as the equivalent human elements. This analysis has shed light on the emergence of these regulatory sequences during early vertebrate evolution, at a time of whole-genome duplications and considerable morphological variation, consistent with a role for these sequences in directing gene regulatory networks for vertebrate development.

## Introduction

Comparisons between diverse vertebrate genomes have uncovered thousands of very highly conserved sequences that show little or no evidence of coding for proteins [1]–[3]. Many of these sequences are found in the proximity of genes that co-ordinate early development, and an increasing number have been shown to function as enhancers in both zebrafish [2] and mouse embryos [4],[5]. Fish-mammal comparisons have allowed the detection of functional elements that are likely to be of importance to all bony vertebrates. Recently, the extraordinary level of conservation displayed by CNEs has been shown to extend through to the Chondrichthyes (sharks, rays and chimaeras) [3] dating these sequences prior to the divergence of cartilaginous and bony fish, over 500 million years ago [6]. Furthermore, the repertoire of CNEs in the genome of the elephant shark (*Callorhinchus milii*) generally encompasses those conserved between mammals and teleost fish [3] indicating that these sequences evolved and became fixed very early in the vertebrate lineage.

By contrast, vertebrate CNEs were not detected at the sequence level in any invertebrate genomes [2], including the urochordates, which are now considered to be the closest relatives to vertebrates [6],[7]. However, analyses suggest that urochordates are evolving very rapidly [6],[8],[9] which may have resulted in loss of CNE-like sequences in this lineage.

More recently, the sequencing of the genome of the cephalocordate, amphioxus (*Branchiostoma floridae*), has uncovered traces of the origins of a very small number of vertebrate CNEs [10],[11], further fuelling speculation that their evolution into a large set of highly conserved non-coding sequences represents a key, defining characteristic of the ancestral vertebrate body plan [2]. Invertebrate groups have been found to possess their own sets of CNEs [12],[13] and interestingly there is a correlation between the classes of genes around which both vertebrate and invertebrate CNEs cluster suggesting parallel evolution of CNE networks [13]. Consequently, whereas the steady evolution of coding sequences can be charted readily across the invertebrate/vertebrate boundary, the evolution of CNEs appears to have been shaped in an entirely different way, with some form of rapid expansion very early in vertebrate evolution. In order to begin to investigate the origin and evolution of CNEs in the early vertebrate lineage we focus here on the lamprey, *Petromyzon marinus*. The lamprey is an agnathan (jawless) fish, and represents, along with hagfish, the earliest diverging vertebrate group, separating from the jawed (gnathostome) lineage between 550 and 650 million years ago [6],[14],[15]. It can be assumed that the common ancestor of agnathans and gnathostomes possessed the developmental mechanisms and characteristics that remain shared by both groups. After their divergence further innovations specific to each lineage would have evolved. As such, comparative studies of the lamprey and its genome will complement existing efforts that examine the evolution of developmental gene regulatory networks [16] and provide valuable insights into the ancestral vertebrate state and the common underlying sequences that determined it.

It is becoming increasingly accepted that the early vertebrate genome was shaped by large-scale/whole genome duplication events, with gnathostomes possessing multiple paralogous copies of genes that are single copy in invertebrates such as amphioxus [10]. The timing of these events is still unclear, but there is evidence to suggest that the emergence of lampreys coincided with one, or both, of the duplications [10] and that the first round occurred before, and the second shortly after, the divergence of lampreys and gnathostomes [17]. However, without an assembled lamprey genome, this is difficult to resolve.

Although most CNEs appear to be unique within the human genome, we recently identified a fraction that are duplicated and found in the vicinity of paralogous genes [18]. Many of these duplicated CNEs (dCNEs) are found in all bony vertebrates and therefore it is likely that they were present (as single copies) in an ancestral genome prior to at least one of the whole genome duplication events early in the vertebrate radiation. This predicts that at least some CNEs might be found in lampreys. It is currently unknown if lampreys have undergone any large scale lineage-specific duplications, although analysis of the HOX cluster suggests that at least this region has duplicated in agnathans independently of gnathostomes [19].

A growing number of CNEs have been tested for their ability to up-regulate gene expression *in vivo* in a number of different animal systems, including zebrafish [2], [18], [20]–[24], medaka [25], mouse [4],[5],[22],[26],[27], chick [28] and *Xenopus* [21]. Generally, a reporter gene under the control of a minimal promoter is injected alongside a CNE, and embryos are screened for tissue specific up-regulation. Results from these assays can then be compared with the endogenous pattern of expression of the gene (usually generated through *in situ* hybridisation) with which the CNE is thought to be associated. Whilst there is a generally good agreement between the two types of expression analyses, the CNE reporter assays often define additional regions of expression not detected for the gene itself [2],[6],[21]. Generally, CNE sequences have been tested in relatively closely related model systems (e.g. human sequences in mouse, Fugu sequences in zebrafish) but given their ubiquity in vertebrates, and to support the notion that they represent a common and fundamental regulatory language, it is critical to establish that CNEs from highly divergent vertebrates function in a similar manner.

Recently, the genome of the sea lamprey, *Petromyzon marinus*, has been targeted for a high quality draft and assembly. As a result, over 18 million whole genome shotgun (WGS) reads, equivalent to approximately 6-fold coverage of the genome, have been made publicly available. Here, we are able to exploit the lamprey data to investigate an ancient epoch prior to, or coinciding with, the emergence of the large repertoire of CNEs currently observed throughout the gnathostome lineage and identify those ancient CNEs that are common to all extant vertebrates. We amplify representative CNEs from both the human and the lamprey genomes, and test them for enhancer activity in zebrafish embryos, thereby assaying regulatory potential spanning over a billion years of evolutionary divergence.

## Results

### Identification and analysis of a set of CNEs from the lamprey whole-genome shotgun sequence

We searched the lamprey whole genome shotgun sequence with a dataset of 1205 CNEs from 13 gene loci that are distributed across 27Mb of the human genome. These regions include 108 duplicated CNEs (dCNEs), the most ancient CNEs for which we have a date of origin [18] and 46 ultraconserved elements (UCEs), sequences that retain 100% identity over at least 200bp between mouse, rat and human genomes [29] (see Methods and Table S1). 73 lamprey CNEs were identified (Table S2), including hits to 38 dCNEs and 14 UCEs, with matches to gnathostome CNEs in all but two of the gene regions (Table 1), implying a widespread distribution of CNEs across developmental regulators in lamprey. Interestingly, the proportion of lamprey hits to dCNEs (38/73 = 52%) is considerably higher than expected (dCNEs only make up 9% of the total CNEs across the 13 regions) demonstrating nearly six-fold enrichment for this particular set of ancient elements (Table 1). Although UCEs are also enriched (30.4% detection rate) it should be noted that this is largely due to the fact that 10/14 of the UCEs are also dCNEs. The high retention rate of dCNEs is of particular note, as it corroborates the notion that an ancient subset of CNEs were present prior to the divergence of the lamprey and gnathostome lineages.

**Table 1:** Conserved non-coding elements from 13 human gene regions identified in the Fugu and lamprey genomes.

			All CNEs			dCNEs			UCEs		
	Human genome		Multi-LAGAN	BLAST hits		Multi-LAGAN	BLAST hits		Multi-LAGAN	BLAST hits	
Gene Region	Chr	Length (Mb)	H/F/M/R	H/F	H/L	H/F/M/R	H/F	H/L	H/F/M/R	H/F	H/L
**BARHL2**	1	0.789	55	49	0	4	4	0	0	0	0
**BCL11A**	2	2.634	72	55	4	6	6	3	8	5	2
**DACH1**	13	1.236	56	50	0	4	3	0	7	4	0
**EBF3**	10	1.753	138	118	9	13	13	3	2	1	1
FOXB1*	15	1.979	45	40	2	5	5	2	0	0	1
**FOXP2**	7	1.682	95	78	8	7	7	2	6	3	1
**IRX5**	16	4.421	192	163	10	20	20	6	3	2	2
**MEIS2**	15	3.079	118	103	10	8	8	4	4	3	1
**NR2F1**	5	3.412	117	98	3	5	5	2	1	1	0
**PAX2**	10	0.264	51	45	9	6	6	5	9	8	5
**TSHZ3**	19	2.194	109	101	10	15	15	8	2	2	1
**ZIC2**	13	0.835	36	29	3	4	4	1	0	0	0
**ZNF503**	10	2.759	121	106	5	11	11	2	4	4	0
**Totals:**		27.039	**1205**	**1035**	**73**	**108**	**107**	**38**	**46**	**32**	**14**

Chr = chromosome, H = human, F = Fugu, M = mouse, R = rat, L = lamprey. H/F/M/R denotes four way multiple alignment (Human/Fugu/Mouse/Rat) used to identify CNEs for further analysis (*FOXB1 alignment uses dog sequence instead of mouse).

The length of the lamprey matches with human CNEs is on average considerably lower than comparisons within gnathostomes (Table S3). Lamprey sequences match on average only 47% of the length of CNEs defined through alignments between mammals and fish using the same BLAST [30] parameters. However, sequence conservation remains high across these shorter core regions, with an average identity of 80%, compared with approximately 90% between fish and mammals (Figure S1 and Figure S2).

### Analysis of a contiguous region containing CNEs from the lamprey genome

**Figure 1:**
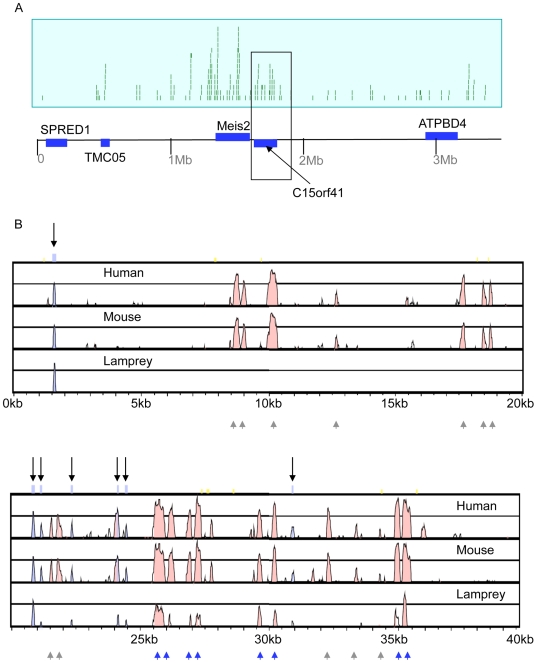
Conservation of non-coding sequences across the Meis2/c15orf41 locus in vertebrates. (A) Plot of non-coding sequence conservation between mammals and fish across a 3.5 Mb region of human chromosome 15q14, encompassing the Meis2 and c15orf41 genes. Each vertical bar within the blue panel represents a CNE. (B) MLAGAN alignment of the c15orf41 locus enclosed by rectangle in (A). Human (Hu), mouse (Mu) and lamprey (La) genomic regions are aligned with the orthologous reigon in the Fugu genome. Exons are annotated and represented by mauve peaks (black arrows) and are detectable in all species. Pink peaks represent non-coding conservation. A number of these are conserved in lamprey (blue arrowheads) but a number are also absent (grey arrowheads).

We searched the pre-Ensembl lamprey release (PMAL3) for assembled contigs that encompass more than one lamprey CNE. The longest (contig 1709) encompasses 33.7kb of contiguous lamprey sequence with just three very short unresolved regions. This contig not only contains a number of CNEs, but it also covers an uncharacterised gene, *C15orf41*, which lies directly downstream of the *Meis2* gene in the human and other vertebrate genomes, oriented in a tail to tail fashion (Figure 1A). The identified lamprey CNEs reside directly adjacent to, or within the introns of, the *C15orf41* gene, and in the human genome form part of a much larger, complex regulatory architecture that covers nearly 3.5Mb of the *Meis2* locus, containing over 200 CNEs (Figure 1A).

Multiple alignment approaches using the conserved coding exons as anchors throughout [31], reveal the organisation of this region, allowing us to identify which gnathostome CNEs are detectable in the lamprey genome. Given the conserved positional relationship of CNEs in all other vertebrates, we assume that if lamprey CNEs are present, they will also be co-linear. From Figure 1B it is apparent that while some CNEs are clearly detectable in the lamprey genome, others are absent, or at least not detectable using sequence similarity. Furthermore, BLAST searches of the WGS reads do not identify these CNE sequences elsewhere in the lamprey genome. The pattern of lamprey CNE occurrence is intriguing; a majority of CNEs across a particular region are present, whereas in the neighbouring region, which harbours some large gnathostome CNEs, no lamprey sequence homology is detected.

### Functional testing of the regulatory potential of lamprey CNEs

One of the most highly conserved CNEs in our data set is found within the sixth intron of the human *EBF3* gene region and extends to 491 bp at greater than 90% identity between Fugu and human. The corresponding region identifiable in the lamprey genome is just 211 bp long (at 79% identity). A second representative CNE, approximately 84kb upstream of the human *PAX2* gene is 85% identical across 425 bp between Fugu and human, but only 123 bp is conserved in lamprey (at 73% identity). We hypothesised that these much shorter but persistently conserved regions of sequence conservation, retained across the extremes of the vertebrate lineage, might comprise critical core *cis*-regulatory modules common to all vertebrates. In order to test this we amplified the core regions from the *EBF3* and *PAX2* CNEs from both the human and lamprey genomes, and used our functional assay [2] to test their ability to up-regulate GFP reporter expression in zebrafish embryos.

**Figure 2:**
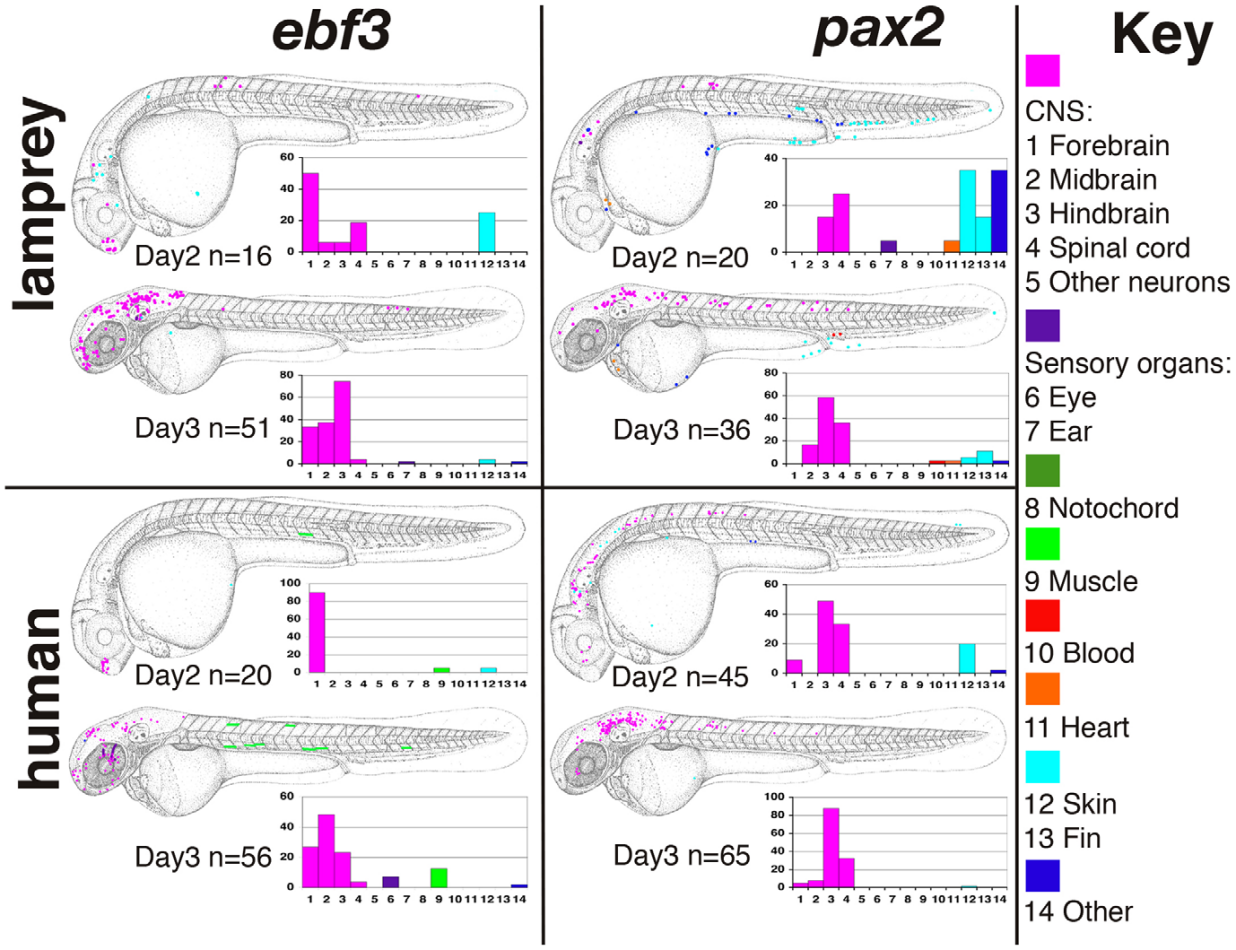
Schematic representations of GFP expression patterns driven by core CNEs. GFP-positive cells are marked onto camera lucida drawings of a zebrafish embryo on day 2 (24–30hpf) and day 3 (48–54hpf) of embryonic development. At each stage, the results are collated from all embryos with expression and overlaid to give a composite depiction of the GFP expression pattern. The number of GFP-positive embryos are noted under each schematic (n = ). The charts show the percentage (y axis) of GFP positive embryos with expression in each domain 1–14 (see below). In both the charts and the schematics, broad domain categories are colour coded. 1, forebrain; 2, midbrain; 3, hindbrain; 4, spinal cord; 5, other neurons (CNS, pink); 6, eye; 7, ear (sensory organs, purple); 8, notochord (dark green); 9, muscle (light green); 10, blood (red); 11, heart/pericardium (orange); 12, epidermal; 13, fin (both light blue); 14, other (dark blue).

**Figure 3:**
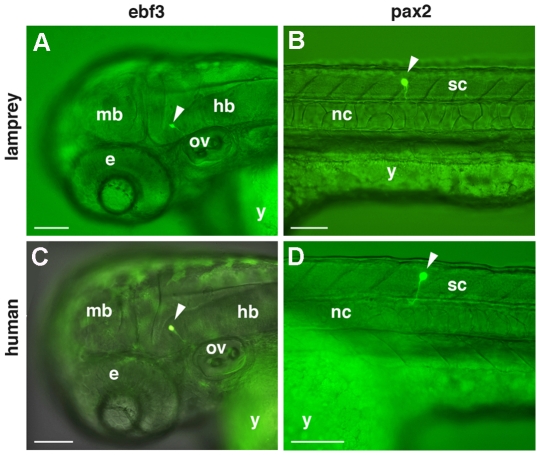
Up-regulation of GFP by lamprey and human derived CNEs. Images of live zebrafish embryos 48–54 hpf (A–C) and 24–30 hpf (D), lateral views, anterior to left. Images are shown as GFP fluorescent (A,B,D) and (C) merged fluorescent and bright field views. (A,C) GFP expression in the hindbrain, driven by an *EBF3* CNE derived from lamprey and human respectively. (B,D) GFP expression in the spinal cord driven by a *PAX2* CNE derived from lamprey and human respectively. e, eye; hb, hindbrain; mb, midbrain; nc, notochord; ov, otic vesicle; sc, spinal cord; y, yolk. Scale bars represent 100 µm.

*EBF3* is a member of the COE (Col-Olf-Ebf) gene family, which consists of the vertebrate orthologues of the *Drosophila collier* gene [32] and the *C. elegans* unc-3 gene [33]. Present as a single copy gene in Amphioxus [34], there are four family members in man and mouse, with considerable conservation of function across animal lineages. *EBF3* is expressed in the developing central nervous system (CNS) [35] and adult brain and recent evidence suggests it acts as a tumour suppressor [36]. Although little is known of the precise function of the *EBF3* gene, it appears to be a key regulator of neurogenesis, associated with the maturation of specific neuronal cell types in the spinal cord and brain [32]. On day two of zebrafish embryo development, at 24–30 hours post-fertilisation (hpf), both the lamprey and human elements direct expression of a GFP reporter gene predominantly in the forebrain, with the human element directing particularly specific expression (Figure 2). However, expression is more widespread on day three, 48–54 hpf, encompassing the spinal cord as well as the fore-, mid- and hindbrain regions. Interestingly, both the lamprey and the human *EBF3* elements appear to up-regulate GFP expression specifically in a particular set of neurons in the zebrafish embryo (Figure 3), demonstrating precise functional conservation between the lamprey element and the equivalent core region of the human CNE, when assayed in a teleost fish model.

*PAX2* is thought to be the most ancient member of the vertebrate *PAX2/5/8* family of genes that arose from early duplications in, or prior to, the vertebrate lineage [37]. It is a transcription factor that plays an important role during the development of the eye, ear, pronephros and midbrain-hindbrain boundary. It is also involved in interneuron specification in the hindbrain and spinal cord [38]. In lamprey, it has been demonstrated that the expression pattern of *pax2* in each region is remarkably similar to that of gnathostomes [39]. Injection of *PAX2* elements derived from both lamprey and human genomic DNA resulted in GFP expression in additional regions to the CNS on day two, but with a more organised pattern of neuronal expression, particularly the hindbrain, on day three (Figure 2). Once again, similar sets of neurons are up-regulated by the human and lamprey elements (Figure 3).

In all four cases the core elements up-regulate GFP expression in a temporal and tissue specific manner that reflects aspects of the endogenous pattern of expression of the associated gene. Furthermore, there is excellent spatial and temporal concordance between the lamprey and human elements.

## Discussion

The ancestry of vertebrate CNEs is clearly identifiable through their abundance in sharks [3], whereas the identification of duplicated CNEs (dCNEs) suggests that some elements were present even earlier [18]. Indeed, within the invertebrates, and despite its earlier radiation from the vertebrate lineage than *Ciona*, the amphioxus genome contains traces of a very small number of CNEs [10]. A total of 56 non-coding, non-repetitive amphioxus sequences were identified with similarity to the human genome [11], displaying on average 64% identity across regions of 50–70bp. Although only a few of these elements overlap with previously identified vertebrate CNEs, they associate once again with genes that regulate development, indicating that the very beginnings of vertebrate CNEs existed in the chordate ancestor of amphioxus and vertebrates. Just 5 of the proposed amphioxus CNEs fall within gene loci covered in our study (3 close to BARHL2, one near BCL11A and one near ZNF503), and none of these have sufficient sequence identity or length to be detected in any vertebrate genome, including lamprey, using unaligned genome wide searches.

Consequently, critical questions remain as to when, how and why such a large repertoire of very highly conserved sequences became fixed in the chordate lineage. Given their proposed regulatory involvement in development, it is essential that there is an understanding of how CNEs evolved and to what extent they contribute to the gene regulatory networks (GRNs) responsible for orchestrating the patterning of the early vertebrate embryo. Unfortunately, there is a dearth of extant organisms that occupy an evolutionary position between the chordate radiation and the emergence of jawed vertebrates. Only lampreys and hagfish survive from this period, and it is therefore both timely and convenient that the genome sequence of the sea lamprey, *Petromyzon marinus*, is being generated. Currently, there is only a limited assembly, yet the public availability of over 18 million sequence traces allows a preliminary foray into the CNE architecture of the genome.

CNEs from 11 out of the 13 regions chosen for this study have matches in the lamprey whole genome shotgun reads, indicating that CNEs are widespread in lamprey. Only the *DACH* and *BARHL2* gene CNEs gave no matches against the lamprey sequence, although there is evidence that *DACH-like* and *BARHL-like* coding sequence is present in lamprey. It is not yet apparent how uniform the WGS sequence coverage is for the lamprey genome although our mapping of over 20,000 lamprey ESTs back to the WGS reads predicts greater than 95% coverage (Methods). Nevertheless, gaps may exist to account for the absence of CNEs around some genes.

It is evident that gnathostome CNEs have evolved extremely slowly, given their near identity between sharks and mammals [3], species which are thought to have diverged over 500 million years ago [6],[15]. This may be a reflection, given their proposed function, of a stable and shared gene/genome copy number and a common bilateral body plan. Indeed, CNEs within the gnathostomes show most variation in teleost fish, a lineage that has undergone its own genome duplication event [40].

**Figure 4:**
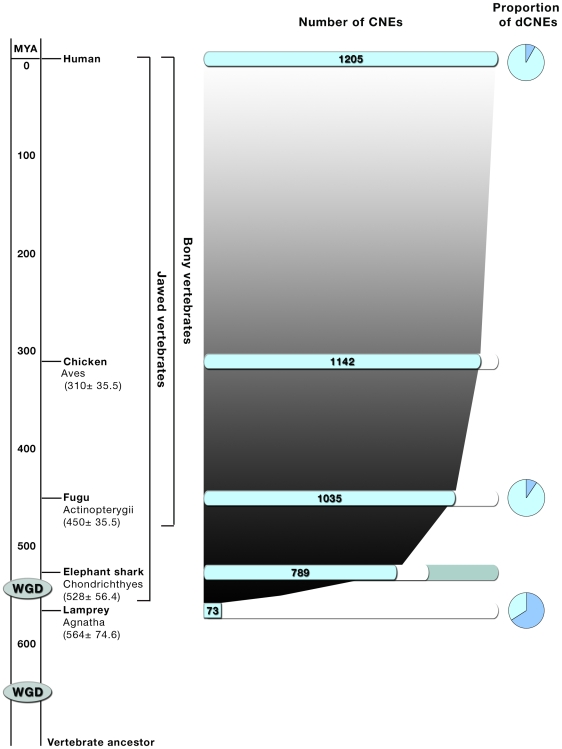
Proposed model of early vertebrate evolution, genome duplications, and expansion of CNE repertoire. Evolution of CNE repertoire occurred early in the history of vertebrates, coinciding with proposed genome duplication events and the emergence of agnathans. CNEs are barely detectable in invertebrate genomes and therefore must have evolved very early in the ancestral vertebrate, coinciding with whole genome duplication (WGD) events. The lamprey genome possesses a much smaller set of CNEs than sharks and other jawed vertebrates. This suggests that a large number of CNEs evolved and became fixed in all gnathostomes within a relatively short time period, between the emergence of agnathans and Chondrichthyes, coincident with the second proposed large scale genome duplication in jawed vertebrates. The proportion of CNEs that are found to be duplicated in the human genome is shown for each species (proportion of dCNEs); over half of the human CNEs that have matches to lamprey are found to be duplicated in the human genome. Timescale and divergence times taken from [15]. The elephant shark genome has a maximum coverage of 75% [3] and the light blue area represents the unsequenced portion.

Lamprey CNEs on the other hand, whilst widespread throughout the genome, are considerably shorter and somewhat less well conserved at the nucleotide level than their gnathostome counterparts (Table S2, Figure S1, Figure S2, Figure S3). This is in part a function of the increased evolutionary divergence between agnathans and gnathostomes but it may also reflect a particularly dynamic and unstable era, early in the vertebrate radiation, during which one or possibly two whole genome duplications occurred. Given their unique developmental characteristics, we suggest that lampreys may have diverged at a time when genomes and CNEs were rapidly evolving, and the vertebrate body plan itself was taking shape (Figure 4). Hence the contemporary repertoire of lamprey CNEs retains only a core set of conserved regulatory signatures that act to specify common features within rather different body plans. This further supports the theory that lampreys separated from the vertebrate lineage prior to at least one whole genome duplication that occurred in the ancestor of all other vertebrates [10],[17].

The strong enrichment for dCNEs in our lamprey data is interesting. Firstly it confirms that dCNEs are ancient, being present in the ancestral vertebrate genome prior to the divergence of lampreys and gnathostomes. The fact that over half the detected lamprey CNE sequences are dCNEs in gnathostomes also supports the notion that the smaller repertoire of CNEs in lamprey is due to the separation of its lineage during a time when CNE sequences were evolving and becoming fixed, such that the stem group of the lamprey lineage only had a relatively small number of CNEs for lamprey to inherit. The alternative scenario, in which all CNE sequences were present in the ancestor to both the agnathans and gnathostomes followed by considerable divergence only in agnathans, struggles to explain the high ratio of dCNEs present in the lamprey genome (i.e. why should the dCNEs not evolve at the same rate as non-dCNEs, thereby preserving the ratio of CNE:dCNE). Additionally, this result suggests that the emergence of many CNEs in vertebrate genomes coincided with, and was perhaps facilitated by rounds of whole genome duplication.

The identification of the *C15orf41*gene contig allows an insight into the CNE landscape of the lamprey genome. At one end of the gene region, a majority of gnathostome CNEs are detectable in lamprey, yet in the other half of this region, there are no lamprey CNEs present. This could indicate that sets of CNEs co-operate locally across relatively large distances in order to function as modules, something that has not been considered to date, although without further examples, it is not possible to draw any more general conclusions.

We chose two very highly conserved CNEs for functional analysis. The first, a CNE near the *EBF3* gene, is over 90% identical across almost 500bp in jawed vertebrates, yet the lamprey identity extends to just over 200bp across the centre of this CNE. We reasoned that this shorter region, given its presence across all vertebrates, might be able to drive reporter gene expression in zebrafish embryos and might therefore define a core region of the human element. Both the human core and the lamprey element drove very specific, near identical, patterns of GFP expression in the developing zebrafish brain, confirming that the shorter region of reduced conservation still retains the basic instructions for this enhancer function. A similar result was obtained for a second CNE, from the *PAX2* region, which shows an even more dramatic reduction in length in lamprey, being less than 30% of the length of the gnathostome CNE. Up to now, the long length and high sequence identity of CNEs has made them recalcitrant to analyses that aim to identify regulatory language encoded within them. The lamprey sequence, combined with functional assays, provides a new angle to this approach and may identify important functional motifs within CNEs.

The shorter regions of identity defined by the lamprey sequences appear, at least in the case of the two elements tested here, to be sufficient to drive a highly specific pattern of reporter gene expression in a limited number of structures. By contrast, the majority of the expression data generated from CNEs defined by fish-mammal comparisons, tends to be less specific and often encompasses a range of tissues [2],[5],[21]. Thus the flanking regions of a gnathostome CNE, not conserved in lamprey, might encode additional functional signatures which have evolved since the agnathan divergence, but which are still common, and therefore conserved, within all bony vertebrates. This suggests that CNEs are multi-functional modules that are built from the middle out, and might explain the unusual size of CNEs for *cis*-regulatory sequences.

CNEs found in common between lampreys and other vertebrates are likely to represent the most ancient regulatory instructions for the ancestral vertebrate body plan. The completion and assembly of the lamprey genome will provide an outstanding resource from which many facets of our vertebrate ancestry may be traced. Investigation of the role and function of lamprey CNEs on a whole genome scale will provide a critical starting point for the building of gene regulatory networks and for understanding the most fundamental language of vertebrate development.

## Methods

### Bioinformatics

In a previous analysis, we identified a set of 256 human-Fugu CNEs that have been duplicated and retained in the vicinity of paralogous genes. Approximately two thirds of these duplicated CNEs (dCNEs) appear to have arisen very early in the vertebrate lineage, pre-dating a whole genome duplication event that occurred before the divergence of fish and tetrapods making them a useful starting point for identifying conserved elements in lamprey. We selected the 13 gene regions containing the largest numbers of dCNEs (3 or more) as identified in [18], and sensitively aligned them using the program Multi-LAGAN [31] using human, mouse, rat (or dog if either mouse or rat not available) and Fugu regions with a window size of 40bp and a cut-off of 65% identity (data available at http://condor.fugu.biology.qmul.ac.uk/). Regions extend to the nearest neighbouring gene, outside of which there are no CNEs. A further filter was applied to eliminate smaller elements by using an “LPC” score of > = 50, a score based on the sequence length and identity across the four species (http://condor.fugu.biology.qmul.ac.uk/tutorial.html#lpc). In order to not have a redundant CNE set (containing both copies of a dCNE), only the region with the highest total number of CNEs from each set of regions containing paralogous genes was selected for further analysis. *SALL1* is included in the *IRX5* region and so was not analysed separately. The resulting 13 gene regions encompass a total of 27 Mb of sequence and contain a total of 1205 elements (Table S1). The 1205 CNEs identified were searched against the chicken and Fugu genomes in Ensembl, and against the lamprey reads (downloaded from the NCBI trace server: http://www.ncbi.nlm.nih.gov/Traces/trace.cgi) using BLAST [30] (-W 8 –q -1 –e 5e-4). A parallel analysis of the lamprey reads using discontiguous MegaBLAST [41] gives essentially identical results. Details of sequence hits can be found in Table S2.

Contamination was suspected in the *Petromyzon* data as some hits were identical to chicken when compared to all vertebrates in Ensembl using BLAST. The full length clones for each of the *Petromyzon* hits were retrieved and compared to the chicken genome using BLAST (default parameters) and reads that had the highest match to chicken, with over 90% identity across most of their length and extending into non-conserved regions were removed (35 lamprey hits).

As the lamprey sequence is not assembled, many lamprey hits were to multiple redundant reads. Consensus sequences were generated for each hit if sequences were more than 95% identical. Overlapping hits were joined to make the longest contiguous hit.

UCEs [29] were selected if they fall within one of the 13 regions, and then searched against the chicken, Fugu and lamprey genomes as for the CNEs using default BLAST parameters. The *Petromyzon* trace data was checked for genome coverage using EST sequences downloaded from NCBI. 20,732 EST sequences were BLAST searched against the trace data using stringent parameters (word size = 25, E-value = 1×10^−10^) and only 969 were found to have no significant hit, indicating that there is approximately 95% coverage.

### Functional assay

Core CNE sequences conserved between human and lamprey for the two selected elements (Figure S3) were PCR amplified from the genomic DNA of each species. This was then purified using QIAquick columns (Qiagen) and co-injected with a GFP reporter into zebrafish embryos. This assay and the subsequent imaging are described previously [2].

## Supporting Information

Figure S1 Example CNE alignment from FOXP2 gene region with regions of 100% identity shaded black.
(0.41 MB DOC)

Figure S2 Percent identity of the lamprey CNE set (73 elements) in various species compared to human (Chimp, dog, mouse, Rat, Opossum, Chick, Frog, Fugu, Tetraodon, Zebrafish, and Lamprey). Only the regions of the multiple alignments that align to lamprey were considered. Mammals generally have >95% identity to human in these regions. It can be seen that the mean percent identity of Lamprey to human is lower than any of the fish but is still above 80%.
(0.09 MB DOC)

Figure S3 Alignments of sequences used in functional analysis. Multi-species sequence alignments around CNEs associated with *EBF3* and *PAX2*. The CNEs defined by mammal-Fugu comparisons are shaded. The core sequence that is also conserved in lamprey is highlighted in yellow. Primer sequences are shown in bold. Human accession numbers for the *EBF3* and *PAX2* CNEs are CRCNEAC00012207 and CRCNEAC00000183 respectively. These and orthologous sequences from other species can be found at http://condor.fugu.biology.qmul.ac.uk/. In the case of *EBF3*, BLAST searches identified four different sequences that matched the human CNE. This alignment shows the two largest lamprey elements. Although both were functionally assayed, the results were so similar that we only show data from one of these (Lamprey_2).
(0.04 MB DOC)

Table S1 Gene regions selected for comparison to lamprey trace data to identify CNEs.
(0.03 MB DOC)

Table S2 Details of sequence matches between Human CNEs and Lamprey whole-genome shotgun reads.
(0.04 MB XLS)

Table S3 Lengths and percent identity of lamprey hits compared to human CNEs.
(0.10 MB DOC)
