# Supplementary figures and images for: Early Evolution of Conserved Regulatory Sequences Associated with Development in Vertebrates
Source: PLoS Genet. 2009 Dec 11;5(12):e1000762. doi: 10.1371/journal.pgen.1000762 (PMC2781166; doi:10.1371/journal.pgen.1000762)

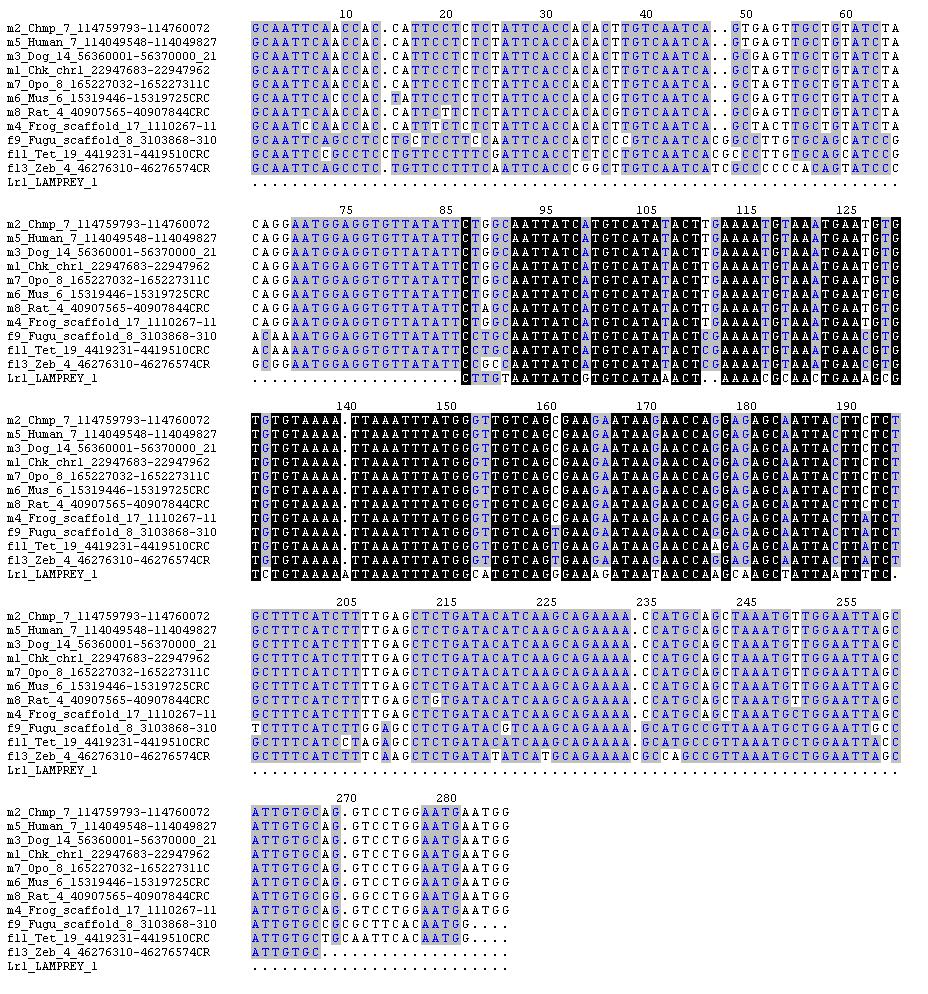


**Figure S1. Example CNE alignment from FOXP2 gene region with regions of 100% identity shaded black.**

Supplement: Figure S1 — Example CNE alignment from FOXP2 gene region with regions of 100% identity shaded black. (0.41 MB DOC) [file pgen.1000762.s001.doc]
