# Supplementary material for: Early Evolution of Conserved Regulatory Sequences Associated with Development in Vertebrates
Source: PLoS Genet. 2009 Dec 11;5(12):e1000762. doi: 10.1371/journal.pgen.1000762 (PMC2781166; doi:10.1371/journal.pgen.1000762)
Supplement: Figure S2 — Percent identity of the lamprey CNE set (73 elements) in various species compared to human (Chimp, dog, mouse, Rat, Opossum, Chick, Frog, Fugu, Tetraodon, Zebrafish, and Lamprey). Only the regions of the multiple alignments that align to lamprey were considered. Mammals generally have >95% identity to human in these regions. It can be seen that the mean percent identity of Lamprey to human is lower than any of the fish but is still above 80%. (0.09 MB DOC) [file pgen.1000762.s002.doc]

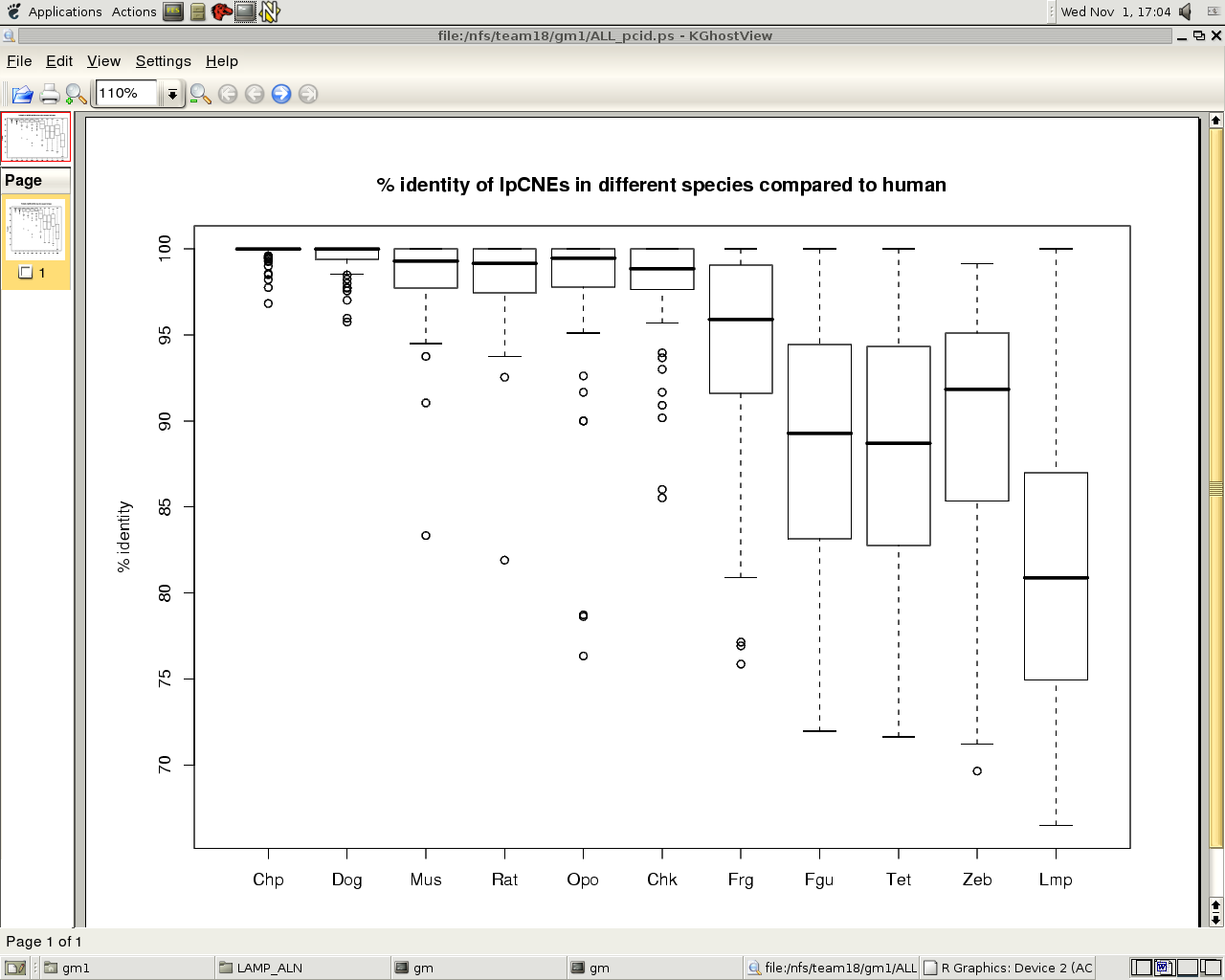


Figure S2. Percent identity of the lamprey CNE set (73 elements) in various species compared to human (Chimp, dog, mouse, Rat, Opossum, Chick, Frog, Fugu, Tetraodon, Zebrafish and Lamprey). Only the regions of the multiple alignments that align to lamprey were considered. Mammals generally have >95% identity to human in these regions. It can be seen that the mean percent identity of Lamprey to human is lower than any of the fish but is still above 80%.
