# Supplementary material for: Early Evolution of Conserved Regulatory Sequences Associated with Development in Vertebrates
Source: PLoS Genet. 2009 Dec 11;5(12):e1000762. doi: 10.1371/journal.pgen.1000762 (PMC2781166; doi:10.1371/journal.pgen.1000762)
Supplement: Figure S3 — Alignments of sequences used in functional analysis. Multi-species sequence alignments around CNEs associated with EBF3 and PAX2. The CNEs defined by mammal-Fugu comparisons are shaded. The core sequence that is also conserved in lamprey is highlighted in yellow. Primer sequences are shown in bold. Human accession numbers for the EBF3 and PAX2 CNEs are CRCNEAC00012207 and CRCNEAC00000183 respectively. These and orthologous sequences from other species can be found at http://condor.fugu.biology.qmul.ac.uk/. In the case of EBF3, BLAST searches identified four different sequences that matched the human CNE. This alignment shows the two largest lamprey elements. Although both were functionally assayed, the results were so similar that we only show data from one of these (Lamprey_2). (0.04 MB DOC) [file pgen.1000762.s003.doc]

**Figure S3. Alignments of sequences used in functional analysis.** Multi-species sequence alignments around CNEs associated with *EBF3* and *PAX2*. The CNEs defined by mammal-Fugu comparisons are shaded. The core sequence that is also conserved in lamprey is highlighted in yellow. Primer sequences are shown in bold. Human accession numbers for the *EBF3* and *PAX2* CNEs are CRCNEAC00012207 and CRCNEAC00000183 respectively. These and orthologous sequences from other species can be found at http://condor.fugu.biology.qmul.ac.uk/. In the case of *EBF3*, BLAST searches identified four different sequences that matched the human CNE. This alignment shows the two largest lamprey elements. Although both were functionally assayed, the results were so similar that we only show data from one of these (Lamprey_2).

**EBF3**

Human -TTTGCAGATTAGCTGGAGAGGCTCGTTCCCGGCGTCAGGAAGTTTGCCAGCAA--GTCT

Mouse -TTTGCGGATTAGCTGGAGAGAC------------TCAGGAACTTTGCCAGCGA--GTCT

Rat -TTTGCGGATTAGCTGGAGAGAC------------TCAGGAACTTTGCCAGCAA--GTCT

Fugu CCTCCCTCCCTCGCCCCTC-GAT------------TCAGGAAGTTTGCCAGCTACTGTCT

Lamprey_1 ------------------------------------------------------------

Lamprey_2 ------------------------------------------------------------

Human TTGTTTACCATGCCAGGGGAAATTGTCAGAGCTGGT---AAAAATTTTCTTCAAAT----

Mouse TTGTTTACCATGCCAGGGGAAATTGTCAGAGCTGGT---AAAAATTTTCTTCAAAT----

Rat TTGTTTACCATGCCAGGGGAAATTGTCAGAGCTGGT---AAAAATTTTCTTCAAAT----

Fugu TTGTTTACAATGCCAAGGGAAAATGTCAGAGCCGTTGAGAAAAATATCCTTAGAATCTTT

Lamprey_1 --------------------------------------------CCGCCGCCACCTCC--

Lamprey_2 ------------------------ATCGATACTCTC--CCGTGAGCTACTTTGGGTAAA**T**

* *

Human -------TTTTTCATC**TTCCTAATCTAACAGTTTACTG**AACTTGATAAGTGTCCTATCAA

Mouse -------TTTTTCATCTTCCTAATCTAACAGTTTACTGAACTTGATAAGTGTCCTATCAA

Rat -------TTTTTCATCTTCCTAATCTAACAGTTTACTGAACTTGATAAGTGTCCTATCAA

Fugu TTTTTTTTTTTTCATCTTCTTAATCTAACAGTTTACTGAACTTGATAAGTGTCCTATCAA

Lamprey_1 -------**TCGCACCGCATTCAGCCG**GCACAGTTTAGCGGACTTGGCCCTCGCCCTATCAG

Lamprey_2 **C------TTGTTTCACTTTCAAGTCAC**TTAGTTTAGCGAACTTGGCCCTCATCCTATCAG

* * * ****** * ***** *******

Human CATGTTAATCAAATTACTTATGAACA-AAAATTGACAGTTTTCATTAATTATACAAGATT

Mouse CATGTTAATCAAATTACTTATGAACA-AAAATTGACAGTTTTCATTAATTATACAAGATT

Rat CATGTTAATCAAATTACTTATGAACA-AAAATTGACAGTTTTCATTAATTATACAAGATT

Fugu CATGCTAATCAAATTACTTCGGAACC-AAAATTGACAGTTTTCATTAATTATACAAGATT

Lamprey_1 CATGTTAATCAAATTACTTCGGAAGGTAAAATTGACAGTTTTCATTAATTATACAGCGCC

Lamprey_2 CATGTTAATCAAATTACTTCGGAAGGTAAAATTGACAGTTTTCATTAATTATACAGCGCC

**** ************** *** ****************************

Human -ATTCTAATTGCAATTCAAATTTATTCATTTAGAACAGAAGGTATTCAGTAATATTTTAC

Mouse -ATTCTAATTGCAATTCAAATTTATTCATTTAGAACAGAAGGTATTCAGTAATATTTTAC

Rat -ATTCTAATTGCAATTCAAATTTATTCATTTAGAACAGAAGGTATTCAGTAATATTTTAC

Fugu -ATTCTAATTGCAATTCAAATTTATTCATTCCGAACAGAAGGTATTCAGTAATATTTTAC

Lamprey_1 GATTCCATTTGCAATGCAAATGGCTTCGTGCGGAACAGAAGGCGCTCAGTAATATTTCAG

Lamprey_2 GATTCCATTTGCAATGCAAATGGCTTCGTGCGGAACAGAAGGCGCTCAGTAATATTTCAG

**** * ******* ***** *** * ********** ************ *

Human AAAATTTGCATATTAAATGGAGGGAGTTGTACATTATGTATGATAATGTATGCACATTTT

Mouse AAAATTTGCATATTAAATGGAGGGAGTTGTACATTATGTATGATAATGTATGCACATTTT

Rat AAAATTTGCATATTAAATGGAGGGAGTTGTACATTATGTATGATAATGTATGCACATTTT

Fugu AAAATTTGCATATTAAATGGAGGGAGTTGTGCATTATGCATGGTAATGTATGCAGAGTTT

Lamprey_1 CAAATTTGCATATTAAATGGAGGGAGCGCTGTATTATGCATGATAATGTATGCGCAGTTT

Lamprey_2 CAAATTTGCATATTAAATGGAGGGAGCGCTGTATTATGCATGATAATGTATGCGCAGTTT

************************* * ****** *** ********** * ***

Human CTTATTTTTAACTTCAGG-**GCCATATGTGGTGC---TGTTGT**CG----------------

Mouse CTTATTTTTAACTTCAGG-GCCATATGTGGTGC---CGTTGTTG----------------

Rat CTTATTTTTAACTTCAGG-GCCATATGTGGTGC---CGTTGTTG----------------

Fugu CTTATTTTTAACTTCGCG-GCCATATGTGGTGT---CGTTGTAG----------------

Lamprey_1 CGGCCT**GGAAAGCCTGCGAACCATAT**GCGATGCGCACGGTGACATAAGACTTCGCGGCGA

Lamprey_2 CCAAC**CCAGAAACACACGAGCCATA**TGTGCTGAGC-TGCTGTTGCAG-------------

* ** * ******* * ** * **

Human ---------GAGCA--GGTGAAAAGTCTGAGTGTGT---TTAA---TTTGCTTGACA--A

Mouse ---------GAGCA--GGTGAAACGTCTGAGTGTGT---TTAA---TTTGCTTGACA--A

Rat ---------GAGCA--GGTGAAACGTCTGAGTGTGT---TTAA---TTTGCTTGACA--A

Fugu ---------GAGCA--GGTGAAAAGTCTAAGTGTGT---TTAA---TTTGCTTGACA--A

Lamprey_1 CTTGTTTGTGAATATTGGCCACGGGGTTGAGTATAAGGAGCAAAGGTGAGCGTGAGCGCA

Lamprey_2 ACTTAAAACGGGCA--GGGGAAGAAAAAAAAGGCGAGCACTAT---CATATTTAAAA--G

* * ** * * * * *

Human ACATTAGGCTGGGGCT-TGTCAAGTGGAGT-----ATAGTGAATGCCAATCTTTATTTAC

Mouse ACATTAGGCTGGGACT-TGTCAAGTGGAGT-----ATAGTGAATGCCAATCTTTATTTAC

Rat ACATTAGGCTGGGACT-TGTCAAGTGGAGT-----ATAGTGAATGCCAATCTTTATTTAC

Fugu ACATTCGGCTGGAGCT-TGTCAAGGGGAGT-----ATTGTGAATGCCAATCTTTATTCCC

Lamprey_1 TCTTTGCACTGCATCCACAGCAGCAGGCGTCAGGAGCTGTGAGTGCCCTCTCATGATTAC

Lamprey_2 TTTTTTCTTTTTGTTC-TCTTACAGACAGTCACTTATAATGAACATGAGTCACTTTCCCT

** * * ** *** *

Human TCCTTATTGATT----ACCCCAAACTCTAA----ACATCTGCATACCTTGTATAAATTTC

Mouse TCCTTATTGATT----ACCCCAAACTCTAA----ACATCTGCATACCTTGTATAAATTTC

Rat TCCTTATTGATT----ACCCCAAACTCTAA----ACATCTGCATACCTTGTATAAATTTC

Fugu TCTTTATTGATT----ACCCCAAACTCTAA----ACATCTGGAGGT--------AAATGG

Lamprey_1 TCAGCGCTAGCCCGAAACATCAATTCTCAAGGTCACCACAGTACACAAAGCAAAGAGAGG

Lamprey_2 TTTATTCTGATG----CCATATGTTAATAA----GCGGTCTCATGGTATGATTAAATATG

* * * ** * * *

**PAX2**

Human CCCTCTCCCGGCGCTGGCGGCCACCGCACTGCAGCGCGGCACCAGCAGTCTATGACAAAA

Mouse CCCTCTCCTGGCGCTGGCGGCCACCGCACTGCAGCGTGGCACCAGCAGTCTATGACAAAA

Rat CCCTCTCCTGGCGCTGGCGGCCACCGCACTGCAGCGTGGCACCAGCAGTCTATGACAAAA

Fugu CACGCTTGGGCTCCTTGTGGCCTACAAACAGAAGCAGGAC-----CAACCAGTGACGAAA

Lamprey ------------------------------------------------------------

Human ACATTATCCTTCTGCTGCTAAACAATCCAATAAACCTCTCCACAGGGAGAAAGTTAACCT

Mouse ACATTATCCTTCTGCTGCTAAACAATCCAATAAACCTCTCCACAGGGAGAAAGTTAACCT

Rat ACATTATCCTTCTGCTGCTAAACAATCCAATAAACCTCTCCACAGGGAGAAAGTTAACCT

Fugu ACATTAGCGTTCTGGTGCTAAACAGTCCAATAAAGCTCTCTACGGAGAAGAACGTAGCCT

Lamprey ------------------------------------GCGGCACGGGGAGAGACGCGGCGC

** * ** ** *

Human CTTGTCTCTTCGGCTGATGCCTTCCCGCCCACGTGACTGCTGCCACAGTAAATTCAAATC

Mouse CTTGTCTCTTCGGCTGATGCCTTCCCGCCCACGTGACTGCTGCCACAGTAAATTCAAATC

Rat CTTGTCTCTTCGGCTGATGCCTTCCCGCCCACGTGACTGCTGCCACAGTAAATTCAAATC

Fugu CTCCTTTCCCCCAACGCTGGCTCTCACACCACGTGAGGGCTGCCACAGTAAATCCAAATC

Lamprey ACGGAT------GAGAGACGCGTGGGGGACACGCGGGGACGGC---AGCAAAA--AA---

*** * * * * **

Human ATTAGTCTCCTCACCCCATCA--------------AAAAA-------**CTTCTATTAGTAA**

Mouse ATTAGTCTCCTCACCCCATCA--------------AAAAA-------CTTCTATTAGTAA

Rat ATTAGTCTCCTCACCCCATCA--------------AAAAA-------CTTCTATTAGTAA

Fugu ATTAGTCTTCTCACTCCGCCATCACTGGGAAGGAGAAAAAAAGTCTGCCTCTATTAGTAA

Lamprey --CGGACGCGCGCGTGCATCA--------AAAGCGAAACA-----------CAACGGG**AA**

* * * ** *** * * * **

Human **TGGAGACC**TCGCCGGAGACCTTCCGCGGAGAGCGCTGCCTTCCCAATTCCCAGTGGAAAA

Mouse TGGAGACCTCGCCGGAGACCTTCCGCGGAGAGCGCTGCCTTCCCAATTCCCAGTGGAAAA

Rat TGGAGACCTCGCCGGAGACCTTCCGCGGAGAGCGCTGCCTTCCCAATTCCCAGTGGAAAA

Fugu TGGAGACCTCGTCAGAGACCTTCCTCTAAGAGCCCTGCCTTCCTAATTCCCACAGGAAAA

Lamprey **CAATCGGCT-ATTAGCGGCAGAGCG**AACGGCGCCCTCCCTTCTC---TCCCGGCGGGGAA

* * * * * * ** ** ***** **** ** **

Human ATTCTGCGCGAAAAACTAATTCTACTTTTAATAATCTGTCATCAGGA-CCAGAGGCAGTC

Mouse ATTCTGCGCGAAAAACTAATTCTACTTTTAATAATCTGTCATCAGGA-CCAGAGGCAGTC

Rat ATTCTGCGCGAAAAACTAATTCTACTTTTAATAATCTGTCATCAGGA-CCAGAGGCAGTC

Fugu ATCCCCAGCAAAAAACTAATTCTACTTTTAATAATCTGTCATCAGGA-CCAGAGGCAGTC

Lamprey ATTCAACCACAAAAACTCATTTTACTTTTAATAATCTGTCAGCAGCA-CCGGCGGCAGCC

** * ******* *** ******************* *** * ** * ***** *

Human ATAAATTATATGGG-AGCGCGTGCGAGAGCGCG--CCA**CAAGAGGGAAATC---AGGGCG**

Mouse ATAAATTATATGGG-AGCGCGTGCGAGAGCGCG--CCACAAGAGGGAAATC---AGGGCG

Rat ATAAATTATATGGG-AGCGCGTGCGAGAGCGCG--CCACAAGAGGGAAATC---AGGGCG

Fugu ATAAATTATGTGGGGAGCATGTGCAGGAGGGGG--CCACCAGAGGCAACTCGGAAGGGGG

Lamprey ATAAATTATACAGCGAGTGTGTGG**GGGAGCGCTATTCACTAGG**GGGAGATC--AACGGGG

********* * ** *** *** * *** ** ** * ** **** *

Human **AA**AAA----TCAAAGCCCAAGTTTGACAAACTTTCCTGAAGATAAATAATAACTTACAAA

Mouse AAAAA----TCAAAGCCCAAGTTTGACAAACTTTCCTGAAGATAAATAATAACTTACAAA

Rat AAAAA----TCAAAGCCCAAGTTTGACAAACTTTCCTGAAGATAAATAATAACTTACAAA

Fugu AAAAAACATTCAAAGCCTGAGTTTGACAAAGTTTGAAGAAGATAAATAATAACTTATCAA

Lamprey AGGAAGGGAGGGAGGAGGGAGGG-CGGAGGGAAGGAAGGGGGTGGGTGGGAGAGGGCGGT

* ** * * * * * * * * *

Human CAG-GATTAGTTGTAAAGTCACGCTCCGGCGCGCAGCCA-----CCGCGGCAATCACGTC

Mouse CAG-GATTAGTTGTAAAGTCACGCTCCGGCGCGCAGCCA-----CCGCGGCAATCATGTC

Rat CAG-GATTAGTTGTAAAGTCACGCTCCGGCGCGCAGCCA-----CCGCGGCAATCATGTC

Fugu CAG-GATTAGTTCTAAAGTCACGGTTGAGTGATCCACCAAGCGGTTTCCACAGCTCTGAA

Lamprey GGGAGGGAAGGAGGCAAGGCAGCGGCGGGAGAGAGAGCGGGAAAGGGGAGAGAGTGGAAG

* * ** *** ** *
