# Supplementary material for: Early Evolution of Conserved Regulatory Sequences Associated with Development in Vertebrates
Source: PLoS Genet. 2009 Dec 11;5(12):e1000762. doi: 10.1371/journal.pgen.1000762 (PMC2781166; doi:10.1371/journal.pgen.1000762)
Supplement: Table S1 — Gene regions selected for comparison to lamprey trace data to identify CNEs. (0.03 MB DOC) [file pgen.1000762.s004.doc]

**Supplementary Material**

Table S1. Gene regions selected for comparison to lamprey trace data to identify CNEs.

| **Gene Region** | **Human Chr** | **Start** | **End** | **Length (bp)** | **Number of elements** | **Number of dCNEs** |
| --- | --- | --- | --- | --- | --- | --- |
| ***IRX3/5/7*** | 16 | 49614462 | 54035441 | 4420980 | 192 | 20 |
| ***EBF3*** | 10 | 129902364 | 131655578 | 1753215 | 138 | 13 |
| ***ZNF503*** | 10 | 75811485 | 78570088 | 2758604 | 121 | 11 |
| ***MEIS2*** | 15 | 33254704 | 36334198 | 3079495 | 118 | 8 |
| ***NR2F1*** | 5 | 90804845 | 94216651 | 3411807 | 117 | 5 |
| ***TSHZ3*** | 19 | 35229337 | 37423768 | 2194432 | 109 | 15 |
| ***FOXP2*** | 7 | 113558372 | 115240492 | 1682121 | 95 | 7 |
| ***BCL11A*** | 2 | 58000381 | 60634217 | 2633837 | 72 | 6 |
| ***DACH1*** | 13 | 70431721 | 71667610 | 1235890 | 56 | 4 |
| ***BARHL2*** | 1 | 90305416 | 91094804 | 789389 | 55 | 4 |
| ***PAX2*** | 10 | 102362654 | 102627091 | 264438 | 51 | 6 |
| ***FOXB1*** | 15 | 57851040 | 59829970 | 1978931 | 45 | 5 |
| ***ZIC2*** | 13 | 99138146 | 99973637 | 835492 | 36 | 4 |
|  |  |  |  |  |  |  |
|  |  |  | Totals: | 27038631 | 1205 | 108 |
