# Supplementary material for: Early Evolution of Conserved Regulatory Sequences Associated with Development in Vertebrates
Source: PLoS Genet. 2009 Dec 11;5(12):e1000762. doi: 10.1371/journal.pgen.1000762 (PMC2781166; doi:10.1371/journal.pgen.1000762)
Supplement: Table S3 — Lengths and percent identity of lamprey hits compared to human CNEs. (0.10 MB DOC) [file pgen.1000762.s006.doc]

**Table S3 Lengths and percent identity of lamprey hits compared to human CNEs**

| **CNE name** | **Lamprey hit** | **Human Length** | **Lamprey length** | **% human length** | **% identity** |
| --- | --- | --- | --- | --- | --- |
| CRCNEAC00024798 | PMAC-bfs52c09.g1 | 75 | 75 | 100.00 | 84.21 |
| CRCNEAC00024809 | PMAC-asq98b05.g1 | 84 | 84 | 100.00 | 91.67 |
| CRCNEAC00012172 | PMAC-azm51a08.b1 | 133 | 132 | 99.25 | 89.47 |
| CRCNEAC00024829 | PMAC-bcc80h07.g1 | 164 | 160 | 97.56 | 83.75 |
| CRCNEAC00003292 | PMAC-bnx75c10.b1 | 89 | 85 | 95.51 | 82.76 |
| CRCNEAC00024800 | PMAC-blu93a01.g1 | 191 | 177 | 92.67 | 62.71 |
| CRCNEAC00016176 | PMAC-aiz50g07.b1 | 248 | 227 | 91.53 | 66.24 |
| CRCNEAC00024797 | PMAC-bhh24h09.g1 | 127 | 116 | 91.34 | 79.31 |
| CRCNEAC00020589 | PMAC-azl77b12.g1 | 189 | 172 | 91.01 | 93.71 |
| CRCNEAC00024820 | PMAC-atv31b03.b1 | 165 | 147 | 89.09 | 88.44 |
| CRCNEAC00011177 | PMACbme63d12.bl | 133 | 111 | 83.46 | 77.68 |
| CRCNEAC00018063 | PMAC-axv41g07.b1 | 132 | 105 | 79.55 | 90.48 |
| CRCNEAC00016158 | PMAC-bma35g11.g1 | 295 | 232 | 78.64 | 70.09 |
| CRCNEAC00020579 | PMAC-atw29a04.b1 | 360 | 283 | 78.61 | 96.14 |
| CRCNEAC00003288 | PMAC-bhl06a08.g1 | 241 | 185 | 76.76 | 65.05 |
| CRCNEAC00008081 | PMAC-bmr71b01.g1 | 280 | 207 | 73.93 | 78.85 |
| CRCNEAC00032799 | PMAC-blt87b03.g1 | 371 | 272 | 73.32 | 71.33 |
| CRCNEAC00003293 | PMAC-avy63e10.b1 | 176 | 123 | 69.89 | 70.73 |
| CRCNEAC00012171 | PMAC-azm51a08.b1 | 110 | 76 | 69.09 | 94.74 |
| CRCNEAC00012204 | PMAC-blk26e04.g1 | 162 | 111 | 68.52 | 75.68 |
| CRCNEAC00024769 | PMAC-alg12d07.b1 | 181 | 117 | 64.64 | 68.38 |
| CRCNEAC00000096 | PMAC-awj40c11.b1 | 437 | 281 | 64.30 | 77.89 |
| CRCNEAC00003299 | PMAC-ana46e09.b1 | 326 | 199 | 61.04 | 69.35 |
| CRCNEAC00012190 | PMAC-bdk60a01.g1 | 290 | 170 | 58.62 | 65.88 |
| CRCNEAC00000217 | PMAC-bmx21g07.g1 | 149 | 87 | 58.39 | 74.71 |
| CRCNEAC00012323 | PMAC-bnt09c05.b1 | 141 | 82 | 58.16 | 70.73 |
| CRCNEAC00008273 | PMAC-axh08a03.b1 | 331 | 192 | 58.01 | 84.38 |
| CRCNEAC00026321 | PMAC-bai40c11.g1 | 264 | 149 | 56.44 | 78.21 |
| CRCNEAC00003285 | PMAC-bfi92a10.b1 | 408 | 226 | 55.39 | 68.56 |
| CRCNEAC00016183 | PMAC-ara38c06.b1 | 410 | 226 | 55.12 | 83.70 |
| CRCNEAC00012325 | PMAC-bni75c09.g1 | 293 | 158 | 53.92 | 67.09 |
| CRCNEAC00023596 | PMAC-awj40c11.b1 | 423 | 222 | 52.48 | 80.63 |
| CRCNEAC00024788 | PMAC-bma18h07.b1 | 311 | 162 | 52.09 | 77.78 |
| CRCNEAC00024807 | PMAC-akj06g02.g1 | 266 | 138 | 51.88 | 82.61 |
| CRCNEAC00000184 | PMAC-ber39b07.g1 | 297 | 153 | 51.52 | 75.32 |
| CRCNEAC00012203 | PMAC-ayh16a07.g1 | 299 | 154 | 51.51 | 72.73 |
| CRCNEAC00011192 | PMAC-aob36g07.b1 | 270 | 139 | 51.48 | 73.76 |
| CRCNEAC00015116 | PMAC-amt30d12.g1 | 228 | 114 | 50.00 | 78.45 |
| CRCNEAC00000430 | PMAC-atu89a06.b1 | 287 | 141 | 49.13 | 80.14 |
| CRCNEAC00011272 | PMAC-bkz54f01.g1 | 137 | 67 | 48.91 | 76.12 |
| CRCNEAC00003201 | PMAC-bha52a06.b1 | 177 | 83 | 46.89 | 77.11 |
| CRCNEAC00012207 | PMAC-acy18e05.b1 | 475 | 214 | 45.05 | 79.44 |
| CRCNEAC00023487 | PMAC-ann29c09.b1 | 120 | 54 | 45.00 | 79.63 |
| CRCNEAC00023592 | PMAC-bod38h08.g1 | 162 | 72 | 44.44 | 72.22 |
| CRCNEAC00000208 | PMAC-bhp24f07.g1 | 297 | 131 | 44.11 | 65.65 |
| CRCNEAC00003286 | PMAC-awz97c03.b1 | 263 | 115 | 43.73 | 66.38 |
| CRCNEAC00017976 | PMAC-agk89g05.g1 | 128 | 54 | 42.19 | 87.04 |
| CRCNEAC00008277 | PMAC-bho87b05.b1 | 237 | 100 | 42.19 | 73.27 |
| CRCNEAC00023538 | PMAC-ams99b08.b1 | 403 | 161 | 39.95 | 81.37 |
| CRCNEAC00003239 | PMAC-azl62d08.b1 | 575 | 225 | 39.13 | 62.83 |
| CRCNEAC00003225 | PMAC-bof30d03.g1 | 162 | 62 | 38.27 | 90.32 |
| CRCNEAC00011202 | PMAC-bmt70g11.g1 | 280 | 106 | 37.86 | 76.85 |
| CRCNEAC00020590 | PMAC-azl77b12.g1 | 153 | 57 | 37.25 | 89.47 |
| CRCNEAC00023546 | PMAC-bbx38b08.b1 | 409 | 152 | 37.16 | 78.95 |
| CRCNEAC00023523 | PMAC-adv45b12.g1 | 180 | 62 | 34.44 | 79.03 |
| CRCNEAC00003196 | PMAC-blb45f05.b1 | 194 | 65 | 33.51 | 84.85 |
| CRCNEAC00026249 | PMAC-bmu69a11.g1 | 330 | 106 | 32.12 | 70.75 |
| CRCNEAC00000437 | PMAC-bdp82e08.g1 | 212 | 68 | 32.08 | 75.00 |
| CRCNEAC00000464 | PMAC-boe54a04.b1 | 194 | 62 | 31.96 | 79.03 |
| CRCNEAC00011209 | PMAC-ayx84d03.b1 | 208 | 65 | 31.25 | 76.92 |
| CRCNEAC00011174 | PMAC-bdn83f10.g1 | 318 | 98 | 30.82 | 79.80 |
| CRCNEAC00024802 | PMAC-bmt81e09.b1 | 239 | 73 | 30.54 | 72.60 |
| CRCNEAC00000173 | PMAC-awz96g01.g1 | 444 | 130 | 29.28 | 66.15 |
| CRCNEAC00023473 | PMAC-bax22c04.g1 | 195 | 55 | 28.21 | 89.09 |
| CRCNEAC00011167 | PMAC-bca14g06.b1 | 219 | 58 | 26.48 | 79.31 |
| CRCNEAC00004218 | PMAC-bfa75h06.b1 | 384 | 98 | 25.52 | 70.41 |
| CRCNEAC00004240 | PMAC-bml03g10.b1 | 174 | 44 | 25.29 | 90.91 |
| CRCNEAC00032873 | PMAC-ave62g11.g1 | 205 | 50 | 24.39 | 82.00 |
| CRCNEAC00012280 | PMAC-bax00d08.b1 | 267 | 63 | 23.60 | 84.13 |
| CRCNEAC00011268 | PMAC-blq62a01.b1 | 335 | 75 | 22.39 | 74.67 |
| CRCNEAC00040393 | PMAC-aha76g12.b1 | 461 | 103 | 22.34 | 78.64 |
| CRCNEAC00000189 | PMAC-bgr89g05.g1 | 448 | 97 | 21.65 | 86.60 |
| CRCNEAC00026219 | PMAC-bfz75g02.g1 | 596 | 116 | 19.46 | 68.10 |
